# *Streptomyces* Bacteria as Potential Probiotics in Aquaculture

**DOI:** 10.3389/fmicb.2016.00079

**Published:** 2016-02-05

**Authors:** Loh Teng-Hern Tan, Kok-Gan Chan, Learn-Han Lee, Bey-Hing Goh

**Affiliations:** ^1^Biomedical Research Laboratory, Jeffrey Cheah School of Medicine and Health Sciences, Monash University MalaysiaBandar Sunway, Malaysia; ^2^Division of Genetics and Molecular Biology, Institute of Biological Sciences, Faculty of Science, University of MalayaKuala Lumpur, Malaysia

**Keywords:** *Streptomyces*, probiotic, aquaculture, fish pathogens, antibiotic resistance

## Abstract

In response to the increased seafood demand from the ever-going human population, aquaculture has become the fastest growing animal food-producing sector. However, the indiscriminate use of antibiotics as a biological control agents for fish pathogens has led to the emergence of antibiotic resistance bacteria. Probiotics are defined as living microbial supplement that exert beneficial effects on hosts as well as improvement of environmental parameters. Probiotics have been proven to be effective in improving the growth, survival and health status of the aquatic livestock. This review aims to highlight the genus *Streptomyces* can be a good candidate for probiotics in aquaculture. Studies showed that the feed supplemented with *Streptomyces* could protect fish and shrimp from pathogens as well as increase the growth of the aquatic organisms. Furthermore, the limitations of *Streptomyces* as probiotics in aquaculture is also highlighted and solutions are discussed to these limitations.

## Introduction

Statistics have revealed that the global aquaculture production continue to increase rapidly without the sign of reaching its peak. Meanwhile, the production from global capture fisheries has stabilized around 90 million tons since the mid-nineties (Mathieson, 2012). According to the United Nations Food and Agriculture Organization report (FAO, 2014), the global aquaculture production achieved another all-time high of 90.4 million tons including the 66.6 million tons of food fish and 23.8 million tons of aquatic algae in 2012 in response to the rising domestic and international seafood demand. Currently, it has been reported that food fish provides an average of one-fifth of total animal protein intake for the world population estimated at 7.3 billion people (Moffitt and Cajas-Cano, 2014). However, major disease outbreaks have been reported within the aquaculture sector in many part of the world due to the increased fish stocking density, over-crowding and lack of sanitary management with the rapid growth of aquaculture. The rapid spread of infections have led to global estimate of disease losses ranges about a quarter billion US$ annually (Bondad-Reantaso et al., 2005). For instance, the viral infections (white-spot syndromes, yellow head disease and taura syndrome) in shrimp industry has cost billions of dollars worldwide (Flegel, 2012; Lightner et al., 2012). Also, bacterial pathogens such as *Vibrio* sp. (*Vibrio harveyi, V. parahaemolyticus, V. campbellii*) caused luminous vibriosis in shrimp farms resulted in 50–100% mortality and vibrio infections in human (Shruti, 2012; Letchumanan et al., 2014; Wang et al., 2015).

Ever since the discovery of penicillin by Fleming in 1928 (Fleming, 1944), antibiotics have played unparalleled roles in disease prevention and treatment for human and animal health and welfare. In addition to the use in human medicine, antibiotics are widely utilized in food animals and aquaculture either as prophylactic or for growth enhancement (Marshall and Levy, 2011). Therefore, antibiotics are extensively used to ensure the development of the intensive and large-scale aquaculture industry. However, the uncontrolled and indiscriminate use of antibiotics has given rise to the emergence of antibiotic resistant bacteria in the aquaculture (Huang et al., 2015; Letchumanan et al., 2015a,b,c) and aquaculture ponds also have been evidenced as reservoirs for antibiotic resistance genes (Tomova et al., 2015; Xiong et al., 2015). These antibiotic resistance genes can be acquired by human and animal pathogens via horizontal gene transfer (Tomova et al., 2015), hence leading to difficulty in the treatment of infectious diseases. Moreover, the recent evidences of residual antibiotics in the cultured organisms could pose a potential health risk to human consumers (Chen et al., 2015; Pereira et al., 2015; Pham et al., 2015).

In order to overcome the continuous emergence of antibiotic resistance pathogens due to abuse of antibiotics in aquaculture, an alternative to antibiotics is urgently needed for disease prevention and treatment and also improvement of quality and sustainability of aquaculture production. Extensive reviews have done indicating that probiotics could be a promising alternative for antibiotics in aquaculture, demonstrating beneficial effects to host by combating diseases, improving growth and also stimulating immune responses of host toward infections (Newaj-Fyzul et al., 2014; Hai, 2015). Therefore, the aim of this review is to provide an insight on the use of the genus *Streptomyces* bacteria as an alternative to antibiotics, being a probiotic in controlling diseases and improving the health and quality of aquaculture production. Furthermore, this review also discusses the prospects and limitations of *Streptomyces* species as a probiotic in aquaculture.

## Probiotics

The term ‘probiotic’ was initially defined as ‘organisms and substances which contribute to intestinal microbial balance’ (Parker, 1974). It was then revised as ‘live microbial feed supplement which beneficially affects the host animal by improving its intestinal microbial balance’ (Fuller, 1989). Meanwhile, in the case of aquatic animals which have much closer interactions with the external environment as compared to the terrestrial organisms, the external environment and feeding have substantial impacts on the microbial status of the aquatic animals. Hence, Verschuere et al. (2000) suggested that a probiotic for aquatic environments should be known as a live microbial adjunct exhibiting beneficial effect on the host by modulating the host-associated or ambient microbial community. Lately, probiotic was described as live, dead or component of a microbial cell that exerts beneficial effect on host by improving disease resistance, growth performance, feed utilization and health status, through the achievement of microbial balance in both host and ambient environments (Hai, 2015). Literatures have showed the possible mode of action of probiotics in aquaculture include (i) growth promoter, (ii) production of inhibitory compounds, (iii) improvement in nutrient digestion, (iv) water quality improvement, (v) enhancement of immune response, and (vi) competition for nutrient (Defoirdt et al., 2007; Martínez Cruz et al., 2012). In order to achieve a probiotic status, the microbes have to fulfill a number of criteria in term of their biosafety and functionality. The desirable characteristics of a potential probiotic include; (i) not harmful toward the host; (ii) ability to survive during transport to the active site; (iii) capability of colonizing and proliferating within the host; (iv) no virulence genes or antibiotic resistance genes (Hai, 2015). The common microorganisms used as probiotics are *Lactobacillus acidophilus, Lactobacillus casei, Bacillus* sp., *Bifidobacterium bifidum*, *Lactococcus lactis* and also the yeast, *Saccharomyces cerevisiae* (Ouwehand et al., 2002; Salamoura et al., 2014). However, less attention has been put on the use of *Actinobacteria* as probiotics in aquaculture despite being widely known as prolific producer for secondary metabolites, particularly the genus (Butler, 2008). The genus *Streptomyces* demonstrated promising results as probiotics (Das et al., 2010; Augustine et al., 2015). This review aim to discuss the prospects of using *Streptomyces* as a probiotic candidate in aquaculture. **Table 1** summarizes all the features and mechanism of actions of the probiotic effects evidenced in the genus *Streptomyces*.

**Table 1:** The probiotic effects demonstrated by *Streptomyces* bacteria through different mechanism of actions.

Features/Mechanism of actions	Probiotic *Streptomyces* bacteria	Outcomes	References
Antagonistic compounds production • Siderophore production	*Streptomyces cinerogriseus* A03 and A05 *Streptomyces griseorubroviolaceus* A26 and A42 *Streptomyces lavendulae* A41 *Streptomyces roseosporus* A45 *Streptomyces griseofuscus* B15	• All the strains positive for siderophore production, detected using CAS-agar • Displayed antagonistic activity toward *Vibrio* species tested (*V. harveyi*, *V. nereis*, *V. fluvialis*, *V. alginolyticus*,*V. parahaemolyticus*, *V. vulnificus* and *V. anguillarum*) ranging from <10 mm to >30 mm inhibition zones • Suggested the ability of the siderophore-producing*Streptomyces* strains controlled the *Vibrio* pathogens by competing for iron in the marine environment	You et al., 2005
• Anti-biofilm and anti-quorum sensing activity	*Streptomyces albus* A66	• Attenuated the biofilm formation of *V. harveyi* with inhibition rate of 99.3% at 2.5% (v/v) • Dispersed the mature biofilm of *V. harveyi* with degradation rate of 75.6% at 2.5% (v/v) • Suggested the anti-biofilm activity demonstrated by*Streptomyces* A66 through the degradation of the quorum-sensing factor *N*-AHSL (*N*-acylated homoserine lactone)	You et al., 2007
• Anti-virulence activity	*Streptomyces* sp. K01-0509	• Produced guadinomine B, a type III secretion system inhibitor of Gram-negative bacteria, including *Vibrio* sp., with IC_50_ at 14 nM	Iwatsuki et al., 2008
• Anti-viral activity	*Streptomyces* sp. AJ8	• Administrated intramuscularly ethyl acetate extract of the secondary metabolite reduced the white spot syndrome virus load significantly (85%) in the *Fenneropenaeus indicus* after third day of injection	Jenifer et al., 2015

Exoenzyme secretion	*Streptomyces* CLS-28 *Streptomyces* CLS-39 *Streptomyces* CLS-45	• All strains showed good proteolytic activity and variable amylolytic and lipolytic activities • Suggested to facilitate the feed utilization and digestion of the host, resulting in increased weight of *Penaeus monodon* when incorporated in the feed	Das et al., 2010

Growth enhancing effect	*Streptomyces fradiae* and *Streptomyces* sp.	• Improved growth of post-larval shrimp *P. monodon* and ornamental fish, *Xiphophorus helleri* • Produced growth-promoting hormone, indoleacetic acid which enhanced growth of *X. helleri*	Dharmaraj and Dhevendaran, 2010; Aftabuddin et al., 2013

Low pH tolerance and intestinal enzymes resistance	*Streptomyces* sp. JD9	• Showed excellent viability at pH 2 • Displayed resistance to pepsin at 3 mg/mL, bile at 0.3% and pancreatin at 1 mg/mL • Demonstrated good survivability in gastrointestinal conditions	Latha et al., 2015

Water quality amelioration	*Streptomyces fradiae* *Streptomyces* sp. *Streptomyces* CLS-28	• Reduced the ammonia level in the water • Increased the total heterotrophic bacterial populations in the water which helped to accelerate the decomposition of waste materials	Das et al., 2006, 2010; Aftabuddin et al., 2013

Single cell protein	*Streptomyces* sp.	• Used as a protein source for host, increased food conversion rate and food conversion efficiency, enhanced growth performance	Dharmaraj and Dhevendaran, 2010; Suguna, 2012; Selvakumar et al., 2013

*In vivo* protection/challenge experiment	*Streptomyces* CLS-28 *Streptomyces* CLS-39 *Streptomyces* CLS-45	Protection of *Artemia* against *V. harveyi* •*V. harveyi* at 10^6^CFU/mL killed all *Artemia nauplii* in 72 h • Addition of *Streptomyces* strains [at 1% (v/v)] increased the survival of *Artemia nauplii* by 67% and adults by 61% after 72 h exposure to *V. harveyi* at 10^6^CFU/mL Protection of *P. monodon* against *V. harveyi* • *V. harveyi* at 10^7^CFU/mL killed 55% of *P. monodon* after 5 days exposure • *Streptomyces* CLS-28 incorporated in the feed (after feeding for 15 days) increased the survival of *P. monodon* by 67% compared to control (without *Streptomyces*) in 5 days exposure	Das et al., 2010

## *Streptomyces* Sp. as Probiotics in Aquaculture

The genus *Streptomyces* (phylum: *Actinobacteria*) are Gram-positive, high G + C (70%) genome content, soil-living bacteria with characterized branching filamentous morphology. *Streptomyces* sp. has been widely recognized as industrially important microorganism due to its potential in producing diverse range of secondary metabolites (Lee et al., 2014b; Ser et al., 2015a,b; Tan et al., 2015) including antibiotics (Lee et al., 2014a), antitumor agents, antiparasitic, immunosuppressive agents, and enzymes (Manivasagan et al., 2013). The production of a variety of wide-spectrum chemical compounds as demonstrated by *Streptomyces* has the advantage of producing potential antagonistic and antimicrobial compounds that can be valuable as probiotics in aquaculture. The ability of producing antagonistic compounds may help the probiotics to compete for nutrients and attachment sites in the host. For instance, the production of bacteriocins (Desriac et al., 2010), siderophores (Lalloo et al., 2010), enzymes (protease, amylase, lipase; Augustine et al., 2015), hydrogen peroxide (Sugita et al., 2007) and organic acids (Sugita et al., 1997) have been documented from the probiotics used in aquaculture. You et al. (2005) reported a *Streptomyces* sp. with siderophores producing activities and suggested that the use of this *Streptomyces* sp. can influence the growth of pathogenic *Vibrio* sp. by competition for iron in the aquatic environment. Siderophores are ferric ion-specific chelating agents with low molecular masses that are usually produced by microorganisms and plants under Fe-limiting conditions (Ahmed and Holmstrom, 2014). Probiotics with the capability of producing siderophores are believed to outcompete the pathogens by limiting the bioavailability of iron and resulting in growth attenuation of the pathogens as iron is essential for growth as well as biofilm formation (Weinberg, 2004). In addition, *Streptomyces* was also evidenced in the production of inhibitory compounds and metabolites involved in the attenuation of biofilm formation, anti-quorum sensing activity (You et al., 2007) and anti-virulence activity in *Vibrio* sp. (Iwatsuki et al., 2008). Besides displaying inhibitory effect on bacterial pathogens in aquaculture, *Streptomyces* also has been report to exhibit anti-viral activity, specifically against the white-spot syndrome virus (WSSV; Jenifer et al., 2015).

*Streptomyces* is primarily saprophytic, living in diverse soil habitats with the development of branching hyphal filaments under conducive environment (Flardh and Buttner, 2009). This unique growth adaptation allows *Streptomyces* in colonization of the solid substrates by adhering and penetrating to gain access on insoluble organic materials in the soil (Flardh and Buttner, 2009). Different hydrolytic enzymes such as amylase, protease and lipase can be produced by *Streptomyces* to break down the insoluble organic materials to provide nutrients for the formation of densely packed substrate mycelium which is reused to fuel the reproductive phase of aerial growth in producing chains of spores (Chater et al., 2010). These unique physiological adaptations of *Streptomyces* are believed to make them as potential probiotics such as the secretion of exoenzymes which may be helpful in facilitating the feed utilization and digestion once they colonize the host intestine in aquaculture. Das et al. (2010) demonstrated that the feed incorporated with *Streptomyces* increased the weight of *Penaeus monodon* shrimp, suggesting that these *Streptomyces* sp. secreted hydrolytic exoenzymes to improve the amylolytic and proteolytic activity in the shrimp digestive tract for more efficient use of the feed. The feed supplemented with *Streptomyces fradiae* isolated from mangrove sediment was also shown to enhance the growth of the post-larval *P. monodon* (Aftabuddin et al., 2013). Besides showing good growth promoting effects in shrimp, all the feeds supplemented with *Streptomyces* was also shown to improve growth performance of the ornamental fish, *Xiphophorus helleri* (red swordtail fish) after 50 days of feeding trial when compared to control without the *Streptomyces* sp. (Dharmaraj and Dhevendaran, 2010). Furthermore, the similar study also showed that the production of growth-promoting hormone, indoleacetic acid by the *Streptomyces* sp. could be contributed to the better growth rate as demonstrated by *Xiphophorus helleri* fed with *Streptomyces* supplemented feeds (Dharmaraj and Dhevendaran, 2010).

The formation of enzymatic digestion, sonic vibration and desiccation-resistant spores demonstrated by *Streptomyces* are also some of the attractive features for this genus of bacteria to resist the harsh environment conditions (McBride and Ensign, 1987), thereby allowing them to retain longer shelf life in the aquaculture ponds before being taken up or to resist the low pH in the gastrointestinal tracts of the animals. However, it should be noted that *Streptomyces* spore is only resistant to moderately high temperature (McBride and Ensign, 1987) as compared to the highly heat resistant endospores of *Bacillus* sp. which is compositionally and physiologically different from the *Streptomyces* spore. Nevertheless, Latha et al. (2015) reported that the *Streptomyces* sp. isolated from fecal sample of chicken showed excellent viability at pH 2, exhibited strong pepsin resistance (at 3 mg/mL) as well as the resistance toward both bile (at 0.3%) and pancreatin (at 1 mg/mL), suggesting that strains from the animal internal cavities would be better in adapting and colonizing the gastrointestinal of the animals. This is also demonstrated by Das et al. (2010) which isolated *Streptomyces* sp. from the sediment of the shrimp culture system able to reach the digestive system of the shrimp, hence allow easier establishment and growth of the probionts in the host. These findings indicate that the spore-forming capacity of *Streptomyces* with high acidity and bile acids tolerance makes them a more practical alternative than those bacteria with non-spore forming capability and further ascertain the potential of *Streptomyces* as probiotic in aquaculture (Das et al., 2010).

The *in vivo* challenge experiment conducted further proved that *Streptomyces* should be spotlighted as probiotics in aquaculture (Das et al., 2010). This study successfully demonstrated the protection effect of *Streptomyces* on both juvenile and adult *Artemia* (15 days old) from *Vibrio* pathogens. The study showed that the *Streptomyces* at 1% concentration (v/v) resulted in higher survival rates than the untreated control group of *Artemia* after challenged with *V. harveyi* or *V. proteolyticus* at 10^6^ CFU/mL. The protective response shown by the study suggests that *Streptomyces* could be administrated to target organisms through bioencapsulation in *Artemia* as a vector for supplementing the beneficial *Streptomyces* probiotics in aquaculture. Bioencapsulation of probiotics in live food such as *Artemia* and rotifers was demonstrated to be more effective in delivery of the probiotics to the digestive tract of the target aquaculture organisms by previous studies (Gatesoupe, 2002; Suzer et al., 2008). The study also further evaluated the efficacy of the *Streptomyces* in protecting the shrimp *P. monodon* from the *Vibrio* pathogens. The feed supplemented with *Streptomyces* sp. CLS-28 for 15 days was found to be exerting protection effect on shrimp *P. monodon* against the 12 h challenge of *V. harveyi* (LD_50_ at 10^6.5^ CFU/mL; Das et al., 2010). A more recent study reported a marine *S. rubrolavendulae* M56 (accession number KJ403746) was shown to exhibit antagonistic activity against all four *Vibrio* sp. including *V. harveyi, V. alginolyticus, V. parahaemolyticus* and *V. fluvialis* in an *in vitro* co-culture experiment (Augustine et al., 2015). In order to confirm the *in vitro* findings, Augustine et al. (2015) demonstrated that the biogranules *S. rubrolavendulae* M56 resulted in lower percentage of mortality of *P. monodon* post-larvae with the reduction of viable *Vibrio* sp. in the culture system after 28 days.

The build-up of ammonia and nitrite level is a major water quality problem which has considerable effects on the health status of the aquaculture livestock due the accumulation of metabolic waste of cultured organisms and the decomposition of the residual feed. The probiotic *Streptomyces* was also found to regulate the microflora of the aquaculture water besides controlling the pathogenic microorganisms and resulted in a better pond conditions. Literature showed that the application of probiotic product did not adversely affect the microflora of aquaculture in turn increased the protein mineralizing and ammonifying bacterial population which help to accelerate the decomposition process of the accumulated wastes materials (Devaraja et al., 2002). Several studies also demonstrated similar results indicating the reduction of ammonia level and increased in the total heterotrophic bacteria in the ponds/tanks treated with the probiotic *Streptomyces* as compared to the control ponds/tanks (Das et al., 2006, 2010; Aftabuddin et al., 2013). These findings suggested that *Streptomyces* could be applied as probiotics which ameliorate the water quality of aquaculture indirectly improve the growth performance and yield of the cultured organisms.

Traditionally, fish meal has been an indispensable ingredient in commercial aquaculture feeds due to its high protein content with excellent amino acid profile and is highly digestible (Gatlin et al., 2007). However, current feed formulations have shifted to other alternative protein source due to the high cost and limited availability of fish meal. Microbial single cell protein of *Streptomyces* is one of the alternative sources of protein and has been utilized and evaluated for better food conversion efficiency and growth for fish (Suguna, 2012; Selvakumar et al., 2013) and shrimp (Manju and Dhevendaran, 1997). Dharmaraj and Dhevendaran (2010) suggested that the use of *Streptomyces* not only showing beneficial effects as probiotic in aquaculture, the incorporation of *Streptomyces* in the feed is also a cost effective approach as the probiotic bacteria replaced around 30–40% of the fish meal used in a feed. The study demonstrated that *Streptomyces* can be a cheaper alternative protein source in the aquaculture feed (Dharmaraj and Dhevendaran, 2010).

## Limitations of *Streptomyces* as Probiotic in Aquaculture

Geosmin and MIB (2-methylisoborneol) are two common semivolatile terpenoid compounds that exhibit earthy/musty taste and odor produced by *Streptomyces* have been known to reduce the palatability of the cultured livestock and negative impact for aquaculture industries (Auffret et al., 2011). These off-flavor compounds are known to be absorbed and bio-accumulated in the gills, skin and flesh of fish up to 200- to 400-folds as compared to the ambient concentration, resulting in lower commercial value of the fish (Howgate, 2004). Many efforts have been shown in literatures for the removal of these earthy odor compounds from water involving the use of powdered activated carbon, ozonation and biofiltration (Elhadi et al., 2004). Among these technologies, ozonation is suggested to effective in this case with the use of *Streptomyces* as the probiotics in aquaculture. Ozone has been known to remove odorants such as geosmin and MIB from water via oxidation (Gonçalves and Gagnon, 2011). A study has demonstrated that the combined effect of ozonation (at 0.3 mg O_3_/L ROC) and probiotic diets (*Bacillus* sp. S11) was able to protect shrimp *P. monodon* from *Vibrio* challenge test without harming shrimp and the probiotic bacteria in the internal system of shrimp (Meunpol et al., 2003).

Furthermore, the risk of lateral gene transfer of antibiotic resistance genes could be an argument against the use of *Streptomyces* as probiotic in aquaculture. Despite that, there are increasing reports on the antibiotic resistance developed by most of the commonly used probiotics such as *Lactobacillus* sp. (Sharma et al., 2015), *Bifidobacterium* sp. and *Bacillus* sp. (Gueimonde et al., 2013). Furthermore, studies also reported that the antibiotic resistance phenotypes displayed by the probiotic *Streptomyces* strains were generally conferred by their intrinsic resistance properties (Das et al., 2010; Latha et al., 2015). Hence, systematic screening for potential antibiotic resistance gene determinants in potential probiotics genome has to be conducted to assess the potential risks and mobility. Furthermore, curative strategies can be valuable tool to remove the genetic element that harbor antibiotic resistance from the relevant probiotic strains (Morelli and Campominosi, 2002; Rosander et al., 2008). For instance, Rosander et al. (2008) successfully demonstrated the protoplast formation curing method able to remove two resistant plasmids from the parent *Lactobacillus reuteri* (ATCC 55730) and without affecting the probiotic properties of the strain. All in all, *Streptomyces* can be one of the interesting probiotics to be further exploited as an alternative to antibiotics in maintaining a sustainable aquaculture.

## Conclusion and Future Work

**FIGURE 1:**
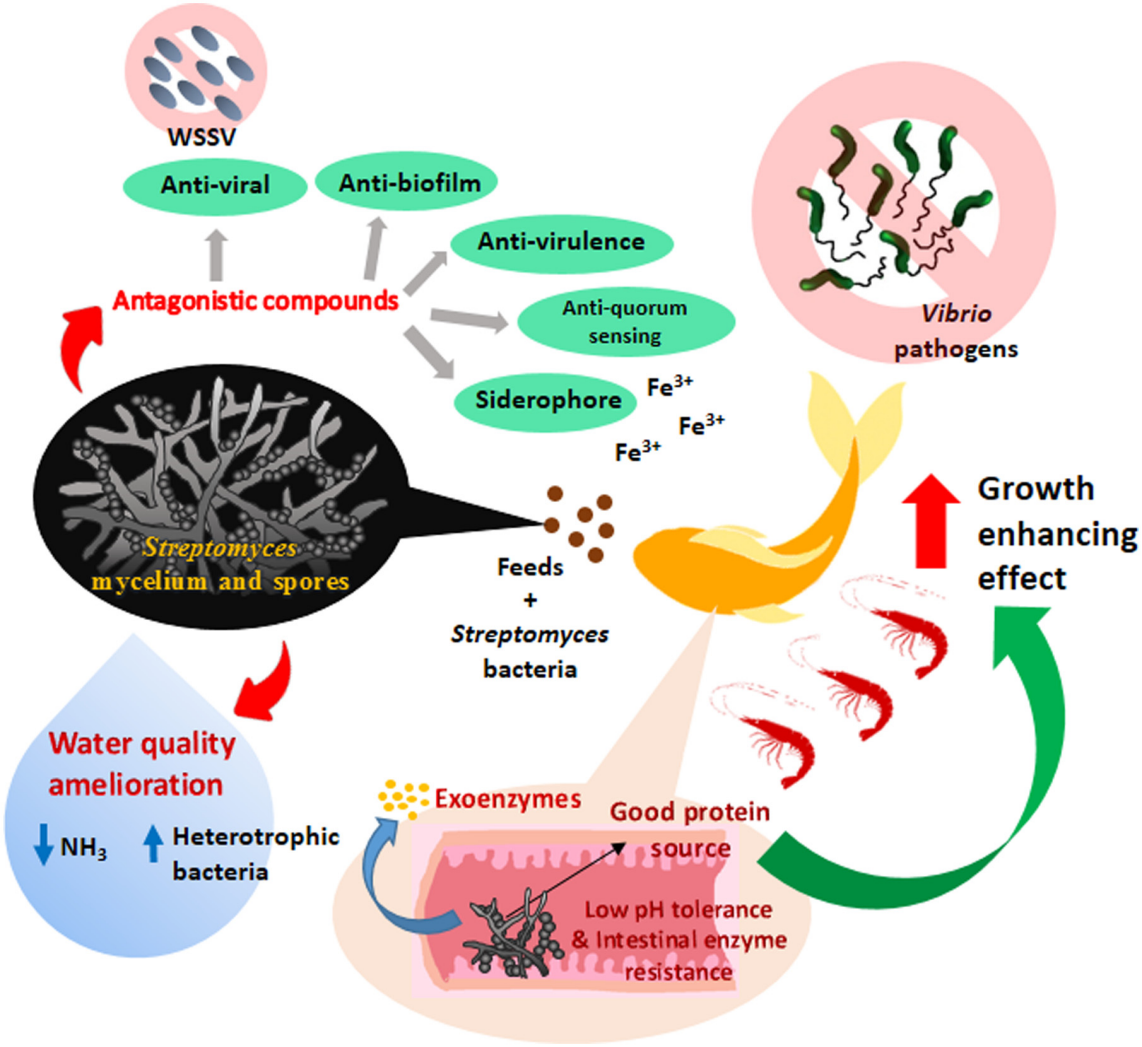
**The mechanism of action of probiotic effects of *Streptomyces* bacteria and their applications in aquaculture.** The *Streptomyces* as probiotic in aquaculture could protect the livestock from pathogens through the production of various antagonistic compounds (e.g., anti-biofilm, anti-quorum sensing and anti-virulence) against *Vibrio* pathogens. *Streptomyces* probiotics attenuate the growth of pathogens by producing siderophores which reduce the bioavailability of iron (Fe^3+^) for the pathogens in the aquatic environment. Anti-viral compounds are also produced by *Streptomyces* probiotic to prevent viral infection caused by white spot syndrome virus (WSSV) in aquaculture. Besides, the *Streptomyces* probiotics also play a role in ameliorating the water quality of aquaculture. *Streptomyces* probiotics help to regulate the microflora, especially to increase the protein mineralizing and ammonifying bacterial populations which accelerate the decomposition process of wastes materials and also ameliorate the water quality by reducing the ammonia level (NH_3_). The consumption of feeds incorporated with the low pH tolerance and intestinal enzymes resistance *Streptomyces* probiotics could enhance the growth performance of the livestock by providing good protein sources. *Streptomyces* probiotics exhibit the ability to secrete hydrolytic exoenzymes which improve the amylolytic and proteolytic activity in the digestive tract of the livestock for more efficient use of the feed; eventually contribute to better growth performance of the livestock.

To date, the number of study employs *Streptomyces* as probiotics in aquaculture is still limited although promising results have been represented by previous studies. A schematic figure is also illustrated to show the mechanism of action of the probiotic effects demonstrated by the *Streptomyces* in aquaculture (**Figure 1**). In order for *Streptomyces* being included among the commonly used biological control agents in aquaculture, further extensive trials are still required to establish the probiotic nature of *Streptomyces* in disease prevention and growth enhancement of aquaculture animals. Furthermore, a better understanding is needed on the exact mode of action of *Streptomyces* involved in probiotic effects. Hence, further research could focus more on molecular techniques to elucidate the possible underlying mechanism portrayed by *Streptomyces* probiotic in aquaculture settings.

## Author Contributions

All authors listed, have made substantial, direct and intellectual contribution to the work, and approved it for publication.

## Conflict of Interest Statement

The authors declare that the research was conducted in the absence of any commercial or financial relationships that could be construed as a potential conflict of interest.
